# Serotonin Receptor and Transporter Endocytosis Is an Important Factor in the Cellular Basis of Depression and Anxiety

**DOI:** 10.3389/fncel.2021.804592

**Published:** 2022-02-24

**Authors:** Nikita Deo, Gregory Redpath

**Affiliations:** ^1^Department of Biochemistry, University of Otago, Dunedin, New Zealand; ^2^European Molecular Biology Lab (EMBL) Australia Node in Single Molecule Science, School of Medical Sciences and the Australian Research Council (ARC) Centre of Excellence in Advanced Molecular Imaging, University of New South Wales, Sydney, NSW, Australia

**Keywords:** depression, anxiety, serotonin receptor, serotonin transporter, endocytosis

## Abstract

Depression and anxiety are common, debilitating psychiatric conditions affecting millions of people throughout the world. Current treatments revolve around selective serotonin reuptake inhibitors (SSRIs), yet these drugs are only moderately effective at relieving depression. Moreover, up to 30% of sufferers are SSRI non-responders. Endocytosis, the process by which plasma membrane and extracellular constituents are internalized into the cell, plays a central role in the regulation of serotonin (5-hydroxytryptophan, 5-HT) signaling, SSRI function and depression and anxiety pathogenesis. Despite their therapeutic potential, surprisingly little is known about the endocytosis of the serotonin receptors (5-HT receptors) or the serotonin transporter (SERT). A subset of 5-HT receptors are endocytosed by clathrin-mediated endocytosis following serotonin binding, while for the majority of 5-HT receptors the endocytic regulation is not known. SERT internalizes serotonin from the extracellular space into the cell to limit the availability of serotonin for receptor binding and signaling. Endocytosis of SERT reduces serotonin uptake, facilitating serotonin signaling. SSRIs predominantly inhibit SERT, preventing serotonin uptake to enhance 5-HT receptor signaling, while hallucinogenic compounds directly activate specific 5-HT receptors, altering their interaction with endocytic adaptor proteins to induce alternate signaling outcomes. Further, multiple polymorphisms and transcriptional/proteomic alterations have been linked to depression, anxiety, and SSRI non-response. In this review, we detail the endocytic regulation of 5-HT receptors and SERT and outline how SSRIs and hallucinogenic compounds modulate serotonin signaling through endocytosis. Finally, we will examine the deregulated proteomes in depression and anxiety and link these with 5-HT receptor and SERT endocytosis. Ultimately, in attempting to integrate the current studies on the cellular biology of depression and anxiety, we propose that endocytosis is an important factor in the cellular basis of depression and anxiety. We will highlight how a thorough understanding 5-HT receptor and SERT endocytosis is integral to understanding the biological basis of depression and anxiety, and to facilitate the development of a next generation of specific, efficacious antidepressant treatments.

## Introduction

Depression and anxiety are the first and sixth highest causes of burden of disability worldwide, respectively (Baxter et al., 2013; Ferrari et al., 2013; World Health Organisation, 2017). Selective serotonin reuptake inhibitors (SSRIs) are the main class of pharmacologic agent used to treat depression and anxiety but are only moderately effective at relieving symptoms (Davey and Chanen, 2016; Cipriani et al., 2018). While up to 30% of patients suffering from depression are resistant to these treatments (Al-Harbi, 2012; Jaffe et al., 2019), SSRIs also have well-established acute anxiety-inducing (anxiogenic) effects (Carvalho et al., 2016). Between 2005 and 2015, the rate of depression and anxiety increased in line with population growth worldwide (World Health Organisation, 2017), highlighting the need for developing more effective new therapeutics for treating these disorders.

Multiple classes of G-protein coupled receptors (GPCRs) have been implicated in the development of affective disorders such as depression and anxiety. GPCRs are common neurotransmitter receptors present on the cell surface throughout the body. GPCRs are coupled to heterotrimeric G proteins consisting of an α, β, and γ subunits which are either activating (β,γ) or inhibitory (α) of downstream signaling responses. Following ligand (such as neurotransmitter) binding to the GPCR, the α and βγ subunits dissociate from the receptor to activate/inactivate intracellular signaling pathways. Dopamine, serotonin, GABA, cholinergic and glutamate receptors have all implicated in mood and mood disorders and the antidepressant response (reviewed extensively in Catapano and Manji, 2007; Senese et al., 2018). Aside from neurotransmitter-binding GPCRs, the orphan class of GPCRs, for which no endogenous ligands have yet been identified, have also been implicated in mood disorders including depression by genetic association studies in humans and knockout/over-expression studies in animal models (reviewed in Orlandi and Watkins, 2020).

Monoamine (neurotransmitters/hormones such as dopamine, serotonin and noradrenaline) transporters are also implicated in the treatment of depression and anxiety, with SSRIs targeting the serotonin transporter (SERT), serotonin noradrenaline reuptake inhibits (SNRIs) targeting both SERT and the noradrenaline transporter (NET) and triple uptake inhibitors targeting all three transporters, preventing the uptake of serotonin, dopamine and noradrenaline into the cell (Lucki and O'Leary, 2004; Zhou, 2004). Other transporters such as ERICH3, which acts as a transporter for loading serotonin into vesicles for release from the cell, have been implicated in antidepressant resistance, with a single nucleotide polymorphism identified in SSRI resistant patients that abolished ERICH 3 transport activity (Gupta et al., 2016; Liu et al., 2020). In this review, we focus on the role the cellular process of endocytosis, serotonin receptors and SERT in the development and treatment of depression and anxiety, but it is likely the endocytic processes we highlight are applicable to many receptors and transporters implicated in the development or treatment of these conditions.

**Figure 1:**
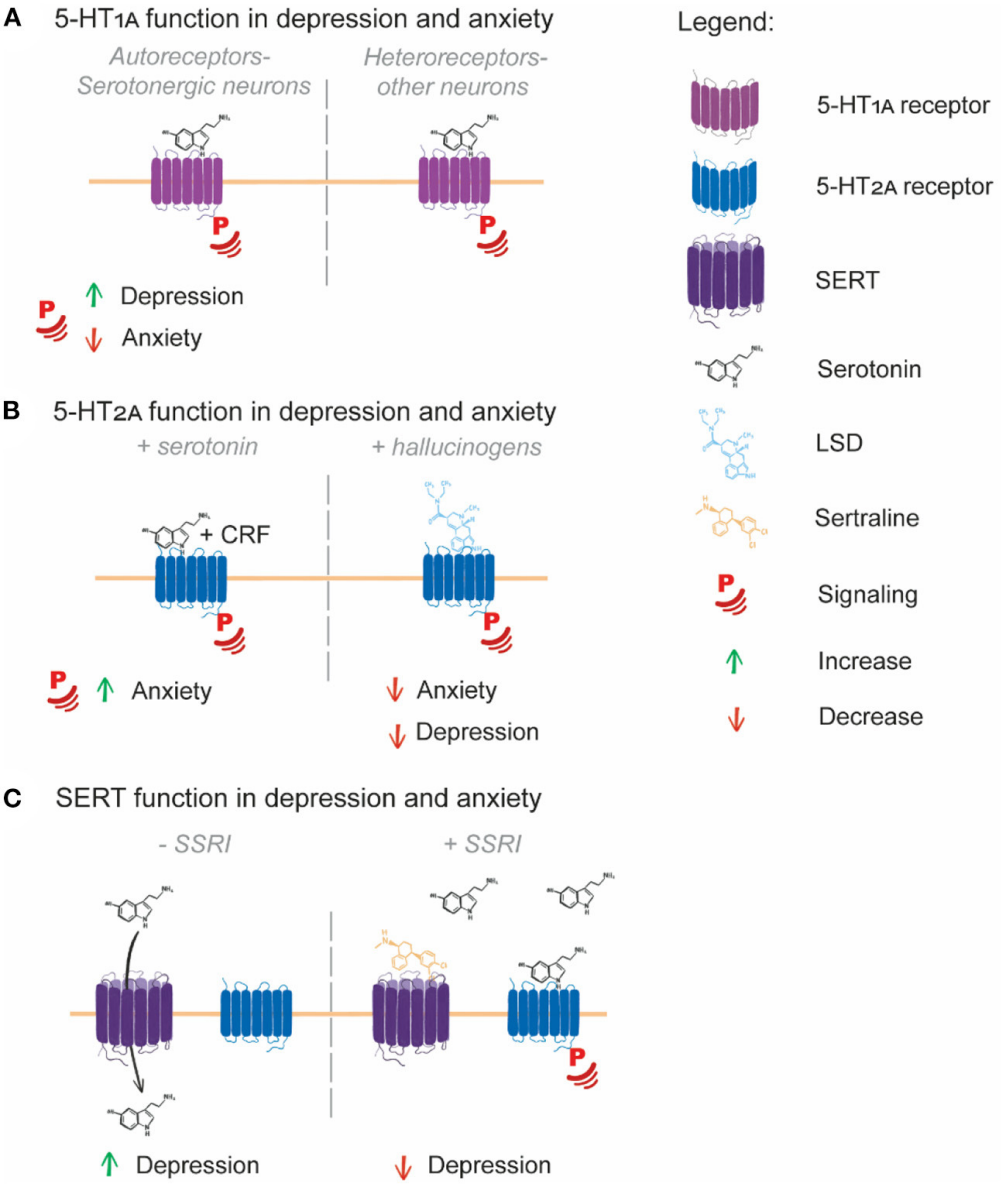
Serotonin system involvement in depression and anxiety. **(A)** 5-HT_1A_ autoreceptors expressed in serotonergic neurons have contrasting roles in depression and anxiety, with increased 5-HT_1A_ signaling potentially increasing depression and decreased signaling increasing anxiety. **(B)** 5-HT_2A_ signaling induced by serotonin and CRF leads to an increased anxiety response in mice, while hallucinogen signaling through 5-HT_2A_ potentially leads to decreased depression and anxiety symptoms in human patients. **(C)** SERT transport of serotonin from the extracellular space to inside the cell limits serotonin receptor signaling, with SERT over-activity potentially being causative of depression. SSRIs block the function of SERT, leading to serotonin accumulation in the extracellular space, enabling enhanced serotonin receptor signaling and alleviation of depression symptoms.

Serotonin is a ubiquitous hormone that is responsible for regulating multiple aspects of mood (Berger et al., 2009) through its action on the serotonin receptors (5-HT receptors). There are seven classes of 5-HT receptor: 5-HT_1_ – 5-HT_7_. All (except, 5-HT_3_) are GPCRs present on the cell surface. Serotonin binding to 5-HT receptors activates the receptor and induces or inhibits intracellular signaling through G-protein signaling, as detailed below. Further regulation of serotonin signaling is exerted by the serotonin transporter (SERT), which induces cellular uptake of serotonin, removing it from circulation, thereby limiting its availability for signaling (Baudry et al., 2019). Changes in or disruption to the serotonin system correlate with depression, anxiety, and their treatment (Figure 1). Most classes of serotonin receptor have been implicated in the development of depression and/or anxiety (Yohn et al., 2017). In this review, we specifically examine 5-HT_1A_, as it has multiple associations with depression and anxiety pathogenesis (Lanzenberger et al., 2007; Kaufman et al., 2016). We will also examine 5-HT_2A_, which is the target of hallucinogens (López-Giménez and González-Maeso, 2017) which are promising candidates for antidepressant and anxiolytic compounds. Finally we will review SERT, the major target of SSRIs (Baudry et al., 2019) (Table 1).

**Table 1:** The relationship between the serotonin system, depression, and anxiety.

**Component**	**Depression**	**Anxiety**	**Treatment**	**References**
5-HT_1A_	Increased receptor binding in the brain, polymorphism can reduce risk	Decreased receptor binding in the brain	SSRIs may decrease autoreceptor levels, alleviating depression but inducing acute anxiety	(Riad et al., 2001, 2004; Lanzenberger et al., 2007; Gray et al., 2013; Zhang et al., 2014; Carvalho et al., 2016)
5-HT2_A_	Potential interaction with serotonin synthesis and depression	Enhances signaling induced via serotonin and corticotrophin-releasing factor induces anxiety in mice	Hallucinogenic compounds induce signaling, antidepressant and anxiolytic effects	(Magalhaes et al., 2010; Yang et al., 2019; Fuentes et al., 2020; Madsen et al., 2020)
SERT	Polymorphisms confer small risk increase, binding availability elevated	Polymorphisms associate with anxiety, binding availability lowered	SSRIs block function, antidepressant and anxiolytic effects	(Meyer, 2007; Reimold et al., 2008; Caspi et al., 2010; Shadrina et al., 2018)
Serotonin	Decreased in peripheral circulation (whole blood, human), increased turnover in brain	Likely increased based upon animal studies	SSRIs decrease whole blood serotonin levels (human), increase brain levels (rodent models)	(Barton et al., 2008; Gupta et al., 2016; Holck et al., 2019)

## Serotonin Receptors, the Serotonin Transporter, Depression, and Anxiety

### 5-HT_1A_ Autoreceptors and Heteroreceptors Have Contrasting Roles in Depression and Anxiety

Two classes of 5-HT_1A_ receptor exist in the human brain: autoreceptors and heteroreceptors (Garcia-Garcia et al., 2014). 5-HT_1A_ autoreceptors are present on neurons synthesizing serotonin (serotonergic neurons) and are responsible for downregulating the serotonin synthesis that occurs in these neurons, while 5-HT_1A_ heteroreceptors are present on non-serotonergic neurons. Developmentally, 5-HT_1A_ autoreceptors help establish the anxiety response (Donaldson et al., 2014; Garcia-Garcia et al., 2014). In platelets isolated from patients with major depressive disorder (MDD, a relatively strictly defined diagnosis for depression), 5-HT_1A_ receptor levels are increased and serotonin levels are decreased compared to controls, with the magnitude of these changes correlating with depression symptoms (Zhang et al., 2014). There is a higher propensity for the 5-HT_1A_ receptor to bind agonists in the brains of people with depression (Parsey et al., 2006) and SSRIs can reduce 5-HT_1A_ receptor binding capacity (Gray et al., 2013). The SSRI fluoxetine specifically targets 5-HT_1A_ autoreceptors over heteroreceptors, inducing their cellular internalization (Riad et al., 2001, 2004), demonstrating that some SSRIs may selectively induce 5-HT_1A_ autoreceptor internalization in the treatment of depression.

SSRI treatments are also noted for their acute anxiogenic effects (Carvalho et al., 2016). In contrast to the brains of depressed individuals, 5-HT_1A_ receptor agonist binding is decreased in the brains of people suffering from anxiety without depressive symptoms (Lanzenberger et al., 2007). Specific deletion of 5-HT_1A_ autoreceptors in the mouse brain gives rise an anxiety response following SSRI treatment, indicating that low or no 5-HT_1A_ autoreceptor signaling is detrimental for anxiety (Turcotte-Cardin et al., 2019). Further, mice expressing a mutant, non-functional 5-HT_1A_ receptor displayed elevated anxiety-like behavior, while also displaying behaviors associated with antidepressant treatment indicating they were perhaps resistant to depression while being prone to anxiety (Heisler et al., 1998). Higher 5-HT_1A_ autoreceptor signaling therefore appears to be a factor in depression, while lower 5-HT_1A_ autoreceptor signaling is a potential factor in anxiety (Figure 1A).

### 5-HT_2A_ Is Targeted by Hallucinogens in the Treatment of Depression and Anxiety

5-HT_2A_ function has not been strongly associated with depression or anxiety, although co-occurring polymorphisms in the serotonin synthesis enzyme TPH2 and 5-HT_2A_ do correlate with MDD (Yang et al., 2019). Interestingly, increased 5-HT_2A_ signaling induced by both serotonin and corticotrophin-releasing factor does induce anxiety in mice (Magalhaes et al., 2010). The 5-HT_2A_ receptor is also a promising target for hallucinogenic therapeutic agents aimed at reducing depression and anxiety. 5-HT_2A_ is potently activated by the hallucinogens lysergic acid diethylamide (LSD) and psilocybin (Almaula et al., 1996; López-Giménez and González-Maeso, 2017), which are emerging as effective antidepressant and anxiolytic treatments in recent clinical trials (Fuentes et al., 2020; Madsen et al., 2020) (Figure 1B). Further exploration of the therapeutic benefits of activated 5-HT_2A_ signaling as a target of depression and anxiety is thus warranted.

### SERT Regulates Extracellular Serotonin Levels

SERT function and SERT polymorphisms are variably linked with depression and anxiety. SERT binding availability is reduced in patients with depression, though reduced binding availability correlates only with severity of anxiety symptoms (Reimold et al., 2008). SERT binding potential however, is elevated in MDD patients (Meyer, 2007), which may indicate that MDD patients have an increased serotonin binding and uptake capacity. Corroborating these data is the finding that a well-established polymorphic region in the SERT promoter region leads to production of more (long allele) or less SERT (short allele) transcript. The short polymorphism has been associated with depression and anxiety, although studies are highly conflicting (Margoob and Mushtaq, 2011), and at most, the short allele confers only a small increase in MDD risk (Shadrina et al., 2018). Interestingly, the association between reduced SERT expression levels with anxiety is present across multiple species, especially when environmental interaction are taken into account (Caspi et al., 2010).

Patients with depression who are SSRI non-responders have lower baseline serotonin levels than depression patients who are responders. Non-responders also have a significantly smaller reduction in whole blood serotonin levels compared to responders (Holck et al., 2019). Since SSRIs are SERT inhibitors, it should follow that SSRIs block serotonin uptake into the cell, raising extracellular levels. However, in peripheral circulation the converse appears to be true: SSRIs reduce total blood serotonin levels (Gupta et al., 2016; Holck et al., 2019). In MDD patients, SSRIs reduce circulating serotonin levels in whole blood, and this reduction correlates with depressive symptom improvement (Gupta et al., 2016). This disparity could be explained by the role that platelets play in serotonin storage. Platelets are a key component of whole blood and a major reservoir of serotonin. Inhibiting platelet-specific SERT with SSRI treatment would likely reduce total serotonin levels in whole blood due to released platelet uptake and storage, which appears to be the case clinically (Karege et al., 1994). Whole blood serotonin is therefore not an adequate indicator of free serotonin available for receptor binding, and the effect of SSRIs are likely reflecting platelet SERT inhibition.

Direct sampling of serotonin levels in the human brain is difficult and impractical. Brain serotonin turnover, as measured by venous blood serotonin metabolite sampling from the internal jugular, is higher in MDD patients compared to controls. This higher turnover was found to be ameliorated by SSRI treatment (Barton et al., 2008). In mice, SSRIs were also found to decrease serotonin turnover, which was attributed to decreased SERT availability for serotonin uptake (Benmansour et al., 2002). Together, these studies indicate that because of the increased serotonin turnover rate in depressed individuals, serotonin levels are likely lower in the brains of patients suffering from depression. SSRI-mediated SERT inhibition then raises brain serotonin levels because of the decreased serotonin turnover (Figure 1C). Together, the serotonin receptors 5-HT_1A_ and 5-HT_2A_ and the serotonin transporter SERT have a demonstrable role in the pathogenesis and treatment of depression and anxiety via their regulation of serotonin signaling and serotonin levels in the brain and periphery. Examining these roles in depression and anxiety indicate that the correct maintenance of 5-HT receptors and the transporter at the cell surface is integral to their functioning. Increased or decreased availability of the receptors/transporters at the surface could therefore be contributing to depression/anxiety pathogenesis.

Endocytosis is the central cellular process that regulates the internalization of receptors and transporters into the cell, thereby regulating their availability for correct functioning at the cell surface (Sorkin and Von Zastrow, 2009). Our understanding of how 5-HT_1A_, 5-HT_2A_ and SERT are endocytosed is in its infancy. In this remainder of this hypothesis and theory review, we will highlight the importance of endocytosis in the regulation of these three components of the serotonin system. Consistent with the importance of endocytosis in their regulation, all antidepressant and hallucinogenic compounds targeting 5-HT_1A_ or 5-HT_2A_ modulate their endocytosis and concomitant signaling responses (Riad et al., 2004; Karaki et al., 2014), and all SSRIs induce SERT endocytosis (Jørgensen et al., 2014). Further, there are a range of genetic, transcriptomic, and proteomic changes identified in patients with depression and anxiety. We hypothesize that these highlighted changes impact the endocytosis of the 5-HT receptors and transporter, and impaired receptor and transporter endocytosis contribute to the biological basis of depression and anxiety.

## Endocytosis and Endosomal Sorting

### Receptor Mediated Endocytosis

Broadly, endocytosis, the process by which cells internalize cargoes such as ligands/receptors/transporters, can be separated into receptor-mediated and receptor independent (fluid-phase) endocytosis. In receptor-mediated endocytosis, a cell internalizes proteins such as receptors and transporters from the plasma membrane following ligand binding. This modulates the internalized receptor/transporters plasma membrane distribution which in turn alters the availability of the respective receptor/transporter for functional outcomes such as activated signaling cascades (Sorkin and Von Zastrow, 2009). Three forms of receptor mediated endocytosis have been relatively well-characterized in mammalian cells: clathrin-mediated endocytosis (CME), fast endophilin mediated endocytosis (FEME) and clathrin-independent carrier/GPI-enriched endocytic compartment (CLIC/GEEC) endocytosis (Redpath et al., 2020; Renard and Boucrot, 2021). In addition to receptor mediated endocytosis, fluid-phase endocytic mechanisms exist, such as macropinocytosis, in which endocytic cargoes are engulfed by the cell (Kerr and Teasdale, 2009). Common to each mechanism of receptor mediated endocytosis is the requirement for a protein coat around the forming endosome, actin remodeling, membrane phosphoinositide conversions and membrane bending BAR-domain containing proteins to induce membrane curvature (Redpath et al., 2020). Fluid phase endocytosis does not require a protein coat, but similarly relies on actin remodeling and phosphoinositide conversions for endocytosis to proceed (Ferreira and Boucrot, 2018). Most relevant to 5-HT receptor (i.e., GPCR) and SERT endocytosis are CME and FEME, while SERT endocytosis potentially occurs via fluid-phase mechanisms upon SSRI treatment (detailed below).

CME is the predominant receptor uptake mechanism in human cells (Bitsikas et al., 2014). When a ligand binds to its receptor, a conformational change is typically induced in the cytoplasmic domain of the receptor. This conformational change allows recruitment and binding of clathrin adaptors such as AP-2 to the receptor (Boucrot et al., 2010). AP-2 binds to phosphoinositide(4,5)P_2_ (PI4,5P_2_) enriched regions of the plasma membrane, initiating clathrin recruitment, leading to formation of a clathrin lattice on the cytoplasmic face of the plasma (Cocucci et al., 2012). Further, actin branching factors localize to PI(4,5)P_2_ enriched membrane regions, which stabilize membrane curvature and assist in endosome extrusion and from the plasma membrane (Redpath et al., 2020). Adaptor localization to the clathrin lattice initiates recruitment of the protein phosphoinositide clathrin assembly lymphoid myeloid leukemia protein (PICALM, or CALM), which drives maturation of the clathrin lattice into an endocytic pit (Miller et al., 2015). Membrane bending BAR-domain containing proteins are also recruited to induce membrane curvature required for lattice maturation, phosphatases are recruited to catabolize the PI4,5P_2_ required for clathrin uncoating following endosome scission, and dynamin recruitment for final scission of the endocytic pit from the plasma membrane (Redpath et al., 2020). Together, these steps result in internalization of receptors or transporters by CME.

FEME is a clathrin-independent endocytic mechanism characterized by the constant formation and dissolution of endophilin “patches” on the plasma membrane (Boucrot et al., 2015). Endophilin is a BAR-domain containing protein, likely involved in the membrane bending required for formation of an endophilin endocytic carrier. In FEME, initially the BAR-domain proteins FIP17 and CIP4 and the actin-remodeling small GTPase CDC42 are recruited to phosphoinositide(3,4,5)phosphate (PI3,4,5P_3_) enriched regions of the plasma membrane, leading to recruitment of the phosphatase SHIP2. SHIP2 metabolizes PI(3,4,5)P_3_ to phosphoinositide(3,4)phosphate (PI3,4P_2_). Endophilin is recruited to and is concentrated at PI(3,4)P_2_ enriched plasma membrane regions, thus forming a primed endophilin patch (Chan Wah Hak et al., 2018). These patches are localized around receptors such as the β1-adrenergic GPCR, and rapidly dissolve if receptor ligand binding does not occur. Where ligand binding does occur, endophilin patches mature into endocytic carriers, actin branching facilitates endosome extrusion from the plasma membrane and dynamin is recruited for scission of the mature endocytic carrier from the plasma membrane, resulting in receptor internalization (Boucrot et al., 2015; Chan Wah Hak et al., 2018).

CLIC/GEEC endocytosis is unique from CME and FEME in that scission of the CLIC/GEEC endocytic carrier from the plasma membrane occurs independent of dynamin (Sathe et al., 2018). Rather, CLIC/GEEC relies heavily on membrane bending by BAR-domain containing proteins and actin branching to facilitate endocytic carrier maturation and scission from the plasma membrane. CLIC/GEEC is activated specifically at the leading edge of a cell by mechanical stimulus (Thottacherry et al., 2018), and has recently been confirmed as a *bona-fide* receptor-mediated endocytic mechanism with the identification of its first cargo-specific adaptor (Moreno-Layseca et al., 2021). To date, CLIC/GEEC endocytosis has not been implicated in GPCR endocytosis.

Macropinocytosis is the predominant mechanism of fluid-phase uptake in the cell. Rather than internalizing a cargo following receptor- ligand binding or involvement of specific endocytic adaptors, macropinocytosis is characterized by extensive plasma membrane ruffling, which internalizes cargo by capturing it and the surrounding extracellular fluid in the ruffled membrane (Kerr and Teasdale, 2009). Macropinocytosis therefore represents a rather non-specific uptake mechanism whereby a plasma membrane-localized protein will be internalized by being captured in the membrane ruffling event. Macropinocytosis can be constitutively active, activated by growth factor receptors or activated by specific cellular conditions such as amino acid starvation (Canton et al., 2016; Charpentier et al., 2020). Unlike the other endocytic mechanisms discussed here, macropinocytosis does not typically rely on endocytic coat proteins such as clathrin, but is rather regulated by a complex series of phosphoinositide conversions that regulate actin remodeling and membrane engulfment (Ferreira and Boucrot, 2018). Constitutively active macropinocytosis is dynamin dependent, while growth factor induced macropinocytosis is dynamin-independent (Cao et al., 2007; Li et al., 2015). While macropinocytosis has not been specifically implicated in GPCR or transporter endocytosis, it is well-established to endocytose plasma membrane proteins as “collateral” during membrane engulfment (Renard and Boucrot, 2021).

### Endosomal Sorting

Following endocytosis of a receptor or transporter, endosomal sorting occurs which controls the receptor/transporter fate and modulates the receptor signaling outcome. Following endocytosis, most endocytic cargoes are endocytosed to the Rab5^+^ sorting endosome, where their endosomal fate is directed (Naslavsky and Caplan, 2018). Four endosomal sorting fates predominate: rapid recycling; constitutive recycling; conditional recycling; and degradation. Rapid recycling to the plasma membrane from Rab5^+^ endosomes is mediated by Rab4 or APPL1, and occurs in a timeframe from seconds to minutes following endocytosis (Yudowski et al., 2009; Jean-Alphonse et al., 2014). Constitutive recycling occurs via Rab11^+^ endosomes, which facilitates continued receptor uptake for nutrient sourcing or sustained receptor signaling and occurs over a timeframe of 5–30 min following endocytosis (Ciechanover et al., 1983; Redpath et al., 2019). Rab5^+^ sorting endosomes mature into Rab7^+^ late endosomes, which subsequently fuse with lysosomes, delivering cargoes for degradation (Rink et al., 2005). A form of conditional recycling can occur from Rab7^+^ late endosomes, which occurs via the retromer/retriever complexes present on Rab7^+^ endosomes (Temkin et al., 2011; McNally et al., 2017). Conditional recycling is modulated by additional stimuli to the receptor ligand, allowing nuanced recycling or degradative responses to regulate processes such as nutrient acquisition or receptor signaling. Conditional recycling via retromer/retriever sequesters cargoes from the late endosome, delivering them to the trans-Golgi network and allowing the cargo to avoid lysosomal degradation (Temkin et al., 2011; McNally et al., 2017).

### G-Protein Coupled Receptor Endocytosis

GPCRs are the largest family of transmembrane proteins in the human genome, and are responsible for transducing ligand binding into cellular signaling for many hormones, neurotransmitters and stimuli (Thomsen et al., 2018). GPCR endocytosis occurs predominantly *via* CME, with a subset endocytosed via FEME (Boucrot et al., 2015). Following ligand binding, GPCRs activate a variety of intracellular signaling pathways via heterotrimeric G-protein binding to the cytoplasmic C-terminus of the receptor (Tsvetanova et al., 2015). In the case of serotonin receptors, serotonin binding to 5-HT_1A_ inhibits adenylate cyclase and cyclic AMP production, neuronal nitric oxide synthase, MEK activation and ERK phosphorylation and T-type calcium channel activation, while activating Rho GTPases, calmodulin, phospholipase C (PLC), phosphoinositide-3-kinase, Src and Ras. Serotonin binding to 5-HT_2A_ activates PLC, PLD, protein kinase C (PKC), adenylate cyclase and cAMP production, endoplasmic reticulum calcium release, calcium channel activation and RhoGTPase activation (reviewed in Masson et al., 2012 and pathway analysis provided in Sahu et al., 2018).

Endocytosis typically serves to limit GPCR signaling. Ligand binding induces phosphorylation of cytoplasmic C-terminal serine or threonine residues by G-protein receptor kinases (GRKs), and less commonly, protein kinase A (PKA) and PKC (Carmona-Rosas et al., 2019; Sulon and Benovic, 2021). GPCR C-terminal phosphorylation by GRKs or PKC allows binding of the proteins β-arrestin1 or 2 (for non-visual GPCRs) to the cytoplasmic C-terminal tail. β-arrestins act as GPCR adaptor proteins, recruiting the clathrin-adaptor AP-2 to the GPCR, leading to translocation of the ligand-bound GPCR to clathrin coated pits or lattices to initiate the clathrin-dependent, dynamin-dependent endocytosis (Laporte et al., 2000; Tsvetanova et al., 2015; Beautrait et al., 2017). C-terminal phosphorylation by GRK, PKC or PKC can also serve to diversify the GPCR signaling response. For example, PKA phosphorylation of the β2-adrenergic receptor lead to receptor retention in the plasma membrane, where it activates L-type calcium channels, while GRK-phosphorylated β2-adrenergic receptor were endocytosed in the same cell and did not contribute to calcium channel activation (Shen et al., 2018). Both GRK and PKC phosphorylation of 5-HT_1A_ and 5-HT_2A_ appears to be important for receptor signaling and internalization, with PKC-mediated C-terminal phosphorylation the best studied mechanism facilitating β-arrestin recruitment in 5-HT_2A_ endocytosis, and the effects of both PKC and GRK on 5-HT_1A_ surprisingly understudied, as detailed below.

Clathrin-independent endocytic mechanisms may serve to diversify the endocytic control of GPCRs. FEME represents a potential β-arrestin independent GPCR uptake pathway, with FEME endocytic carriers having been demonstrated to be devoid of β-arrestins (Boucrot et al., 2015). The β1-adrenergic receptor and dopamine receptor 4 are both internalized independently of β-arrestins and β1-adrenergic receptor is an established FEME cargo, while the dopamine receptor 4 is an interaction partner of endophilin family proteins (Shiina et al., 2000; Boucrot et al., 2015; Xu et al., 2019). Together, this indicates that for specific GPCRs, uptake can be β-arrestin- and clathrin-independent, and potentially mediated by FEME.

### G-Protein Coupled Receptor Endosomal Signaling

A subset of GPCRs can be classified as class-A and class-B based on the affinity of their β-arrestin interaction (Nguyen and Lefkowitz, 2021), and provide an example of how β-arrestin affinity can modulate GPCR endosomal sorting and signaling. Class-A GPCRs have lower affinity for β-arrestin and are rapidly recycled to the plasma membrane following their ligand-induced internalization. Rapid GPCR recycling allows constant endocytosis and recycling, facilitating rapid, iterative signaling responses to continued GPCR ligand binding (Seachrist et al., 2000). The β2-adrenergic receptor undergoes this kind of rapid GPCR recycling, which occurs via Rab4-depedent pathways (Yudowski et al., 2009). Class-B GPCRs have a higher β-arrestin affinity than class-A GPCRs. β-arrestin binding to the GPCR C-terminus was previously thought to terminate or limit GPCR signaling by sterically interfering with G-protein binding. However, significant physiologically relevant signaling occurs from endosome localized, β-arrestin-bound GPCRs (Thomsen et al., 2016), conferring a spatiotemporal aspect to GPCR signaling within the cell. Class-A GPCRs exhibit lower levels of endosomal signaling, despite the lower β-arrestin affinity theoretically allowing for more G-protein binding and signaling pathway activation. Class-B GPCRs can exhibit more robust endosomal signaling, with the bound β-arrestin forming a conformation that facilitates sustained G-protein binding to facilitate signaling from the endosome (Nguyen et al., 2019). The relative affinity of a GPCR for β-arrestin is therefore an important factor determining the GPCR endosomal sorting fate and extent of endosomal signaling.

Endosomal signaling diversifies the GPCR signaling response, conferring additional cellular outcomes beyond those encoded by plasma membrane signaling. For example, initial ligand binding to the parathyroid hormone (PTH) receptor induces transient production of cyclic-AMP (cAMP) at the plasma membrane via G-protein signaling, while also activating β-arrestin binding and receptor internalization. Following internalization, cAMP production is sustained from PTH receptor endosomes (White et al., 2020). cAMP rapidly diffuses within the cell, meaning cAMP produced prior to endocytosis is unlikely to function far beyond the plasma membrane environment. Endosomal cAMP production occurs in the perinuclear region, inducing protein kinase A (PKA) translocation into the nucleus and eliciting a transcriptional response to GPCR signaling (Peng et al., 2021). In contrast, plasma membrane cAMP production induces PKA phosphorylation of the endosomal protein APPL1, which is present on early endosomes within close proximity to the plasma membrane, and this phosphorylation facilitates rapid GPCR recycling (Sposini et al., 2017).

## Serotonin Receptor and Transporter Endocytosis

**Figure 2:**
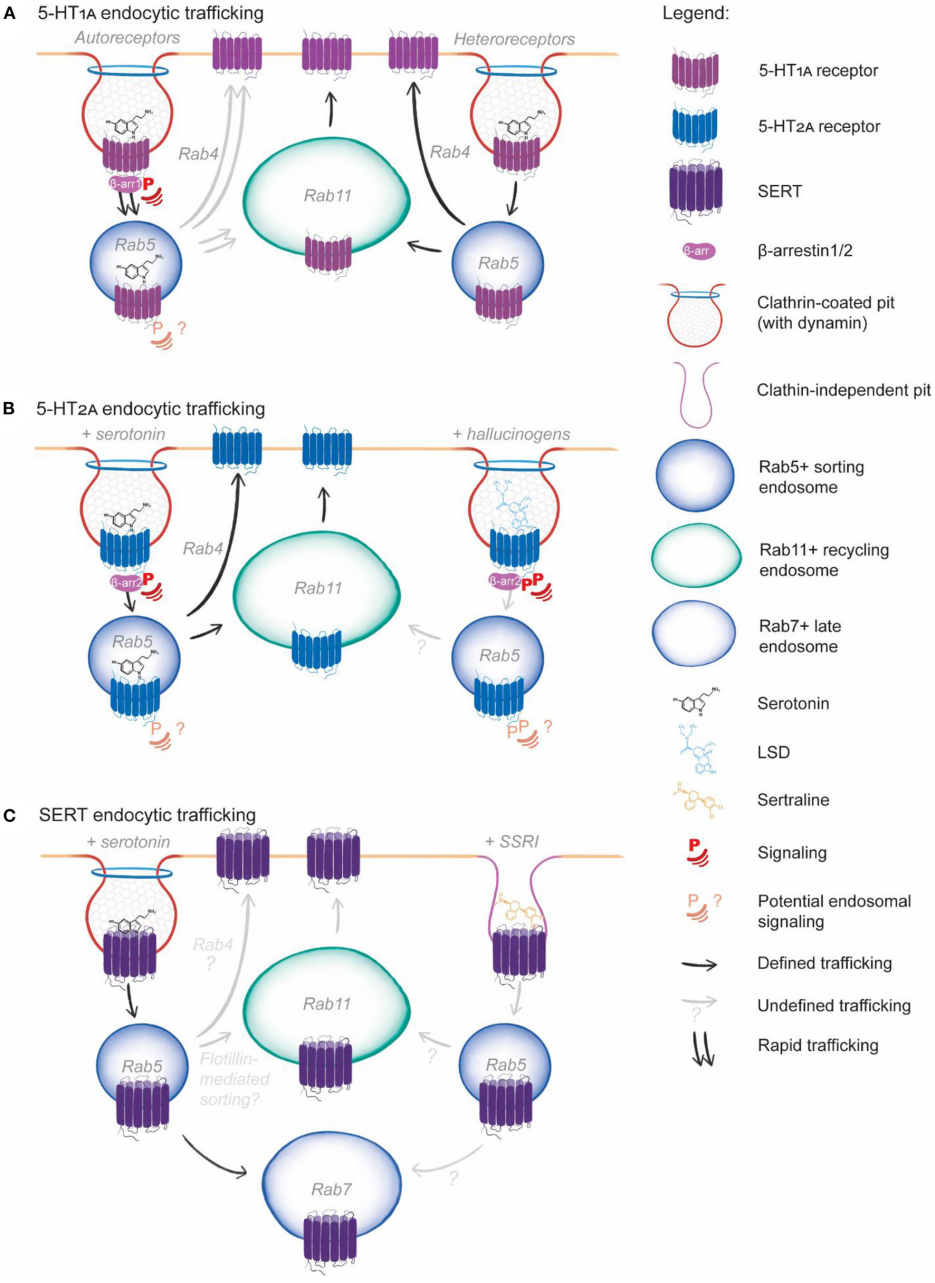
Serotonin system endocytic trafficking. **(A)** 5-HT_1A_ endocytosis is induced by serotonin and is clathrin, dynamin and β-arrestin1 dependent for both autoreceptors and heteroreceptors. Following endocytosis, 5-HT_1A_ is recycled via Rab4 and Rab11-depednent pathways, (this has not been directly demonstrated for 5-HT_1A_ autoreceptors but is consistent with available data, grayed arrow). 5-HT_1A_ autoreceptor endocytosis is induced by serotonin, the SSRI fluoxetine and other pharmacological compounds and leads to rapid endocytic trafficking (double arrows), while 5-HT_1A_ heteroreceptor endocytosis is slower and induced only be serotonin. Endosomal signaling has not been directly demonstrated for 5-HT_1A_ but appears to occur based on pharmacological studies (question mark). **(B)** 5-HT_2A_ endocytosis is clathrin, dynamin and β-arrestin2 dependent in response to both serotonin and LSD binding. Serotonin and LSD induce differential phosphorylation at the plasma membrane. Following serotonin induced endocytosis, 5-HT_2A_ endosomal sorting leads to Rab4 and Rab11-dependent recycling. The effect of LSD on 5-HT_2A_ endosomal signaling and sorting is unknown (grayed arrow, question marks). **(C)** SERT endocytosis is presumable clathrin-dependent and demonstrated to be dynamin-dependent. Following endocytosis, SERT can be sorted to the Rab7^+^ late endosome for lysosomal degradation or can potentially be sorted for Rab11-dependent recycling via interaction with flotillin. The SSRI sertraline induces dynamin-independent endocytosis of SERT, following which the endosomal sorting is unexplored (grayed arrow, question mark).

As GPCRs, 5-HT receptor endocytosis is central to mediating the signaling response to serotonin. Serotonin induces endocytosis of most 5-HT GPCRs (Ponimaskin et al., 2005; Janoshazi et al., 2007; Bohn and Schmid, 2010; Renner et al., 2012; Kumar et al., 2019), as well as endocytosis of its transporter, SERT (Jørgensen et al., 2014) (Figure 2; Table 2). The current state of knowledge of 5-HT_1A_, 5-HT_2A_ and SERT endocytosis and the effects of serotonin and pharmacological compounds on endocytosis is summarized below. Notably there are cell type differences in 5-HT receptor endocytosis and in the induction of endocytosis and the subsequent signaling in response to pharmacological agents and ligands. The final section highlights the missing pieces in our understanding of 5-HT receptor and transporter endosomal trafficking.

**Table 2:** Serotonin receptor and transporter endosomal pathways and agonist effects.

**Component**	**Endocytic pathway**	**Sorting pathways**	**Serotonin effect**	**Therapeutic agent effect**	**References**
5-HT_1A_	CME, β-arrestin1, dynamin	Rab4 and Rab11 recycling	Induces endocytosis (neurons), no effect (kidney)	Fluoxetine: induces endocytosis. 8-OH-DPAT: induces endocytosis	(Riad et al., 2004; Bouaziz et al., 2014; Mondal et al., 2019; Kumar and Chattopadhyay, 2021)
5-HT2_A_	CME, β-arrestin2, dynamin	Rab11 recycling, Rab4 recycling with CRF	Induces endocytosis	DOI, LSD: low endocytosis induction. Lisuride, ergotamine: potent endocytosis induction	(Gray et al., 2001; Nicole et al., 2002; Magalhaes et al., 2010; Baldys and Raymond, 2011; Karaki et al., 2014)
SERT	Likely CME, dynamin	Degradation, potential recycling	Induces endocytosis	SSRIs: induce endocytosis, potentially clathrin independent.	(Kittler et al., 2010; Jørgensen et al., 2014; Rahbek-Clemmensen et al., 2014; Quinlan et al., 2020)

### 5-HT_1A_ Endocytosis

5-HT_1A_ endocytosis is induced by serotonin binding, which induces β-arrestin1-mediated and clathrin-mediated endocytosis. Dominant-negative β-arrestin1 or dynamin-1 expression (Della Rocca et al., 1999), or treatment with the clathrin inhibitor PitStop2 (Mondal et al., 2019; Kumar and Chattopadhyay, 2021), reduces 5-HT_1A_ endocytosis by ~50% following serotonin addition in HEK293 cells. Further, in the mouse neuronal Neuro-2A cell line, 5-HT_1A_ endocytosis occurred robustly in serum-free media and occurred over minutes, indicating CME, and not rapid, serum-dependent uptake via FEME (Casamento and Boucrot, 2020), is the predominant mechanism of 5-HT_1A_ endocytosis. Acute ethanol exposure reduced the internalization of 5-HT_1A_ in Neuro2A cells by inducing the degradation of β-arrestin2, but not of GRK2, indicating that 5-HT_1A_ endocytosis may be independent of GRK2 activity (Luessen et al., 2019). While both PKC and GRF activity are required for signaling outcomes of 5-HT_1A_ in response to the 5-HT_1A_/5-HT_7_ agonist 8-OH-DPAT in RN46A cells, with mutation of PKC phosphorylation sites and over-expression of the C-terminus of GFR2 impeding agonist induced calcium release and MAPK inhibition, however receptor endocytosis was not explored in this study (Kushwaha and Albert, 2005). Further, both studies did not use serotonin as a receptor agonist. GRK and PKC are likely important for 5-HT_1A_ internalization and signaling, but their precise roles are yet to be elucidated.

In both HEK293 (Kumar et al., 2019) and mouse Neuro-2A neuronal cells, 5-HT_1A_ co-localizes with Rab4 and Rab11 (or the Rab11 pathway marker Rab coupling protein, RCP) (Fichter et al., 2010). Rab4 co-localization is also robust in both cell lines almost immediately following endocytosis, and peaks within 10–15 min following serotonin addition. Co-localization of 5-HT_1A_ with Rab11 (or RCP) however, peaks from 30 to 90 min following serotonin addition. No colocalisation with lysosomal compartment markers was observed in either cell line (Fichter et al., 2010; Kumar et al., 2019) indicating that 5-HT_1A_ likely undergoes both fast Rab4-dependent recycling for rapid desensitization/resensitization cycles, and Rab11-dependent recycling for sustained redelivery to the plasma membrane.

5-HT_1A_ endocytosis is differentially induced by ligands and agonists between dorsal raphe nucleus neurons (serotonergic, 5-HT_1A_ autoreceptors) and hippocampal neurons (non-serotonergic, 5-HT_1A_ heteroreceptors) (Figure 2A). In both cell types, 5-HT_1A_ undergoes a basal level of constitutive endocytosis (Bouaziz et al., 2014). Addition of serotonin, the closely related tryptophan derivative 5-carboxamidotryptamine, and the agonist 8-OH-DPAT to serotonergic neurons induce robust 5-HT_1A_ endocytosis following 1- and 24-h treatments, which is abolished with co-incubation of a 5-HT_1A_ antagonist. Hippocampal neurons do not respond to the agonist 8-OH-DPAT, and 5-HT_1A_ endocytosis is only induced by serotonin and carboxamidotryptamine with 24 h of treatment. Further, a subpopulation of hippocampal neurons show no response to any treatments in regard to 5-HT_1A_ endocytosis (Bouaziz et al., 2014). The SSRI fluoxetine induces 5-HT_1A_ endocytosis in serotonergic neurons, but not hippocampal neurons, in the absence of serotonin (Riad et al., 2004). Together, these studies indicate that 5-HT_1A_ autoreceptors (in serotonergic neurons) and heteroreceptors (on non-serotonergic neurons) respond differently to physiological and pharmacological agonists.

A recent study by Sniecikowska et al. (2020) has identified new 5-HT_1A_-specific antidepressant compounds, highlighting the importance of endosomal signaling through 5-HT receptors in regards to developing novel antidepressants. “Compound 44” induced potent ERK activation, while inducing β-arrestin recruitment to 5-HT_1A_ and cAMP production at a level comparable to serotonin. Intriguingly, compound 44 was an effective antidepressant in mice, indicating that specific 5-HT_1A_ signaling profiles are beneficial for alleviating depression. “Compound 56” potently induced β-arrestin recruitment compared to serotonin or compound 44, and increased ERK activation and cAMP production to a much greater extent than compound 44. Compound 56 also acted as an antidepressant in mice, but also led to serotonin syndrome (i.e., serotonin overdose) (Sniecikowska et al., 2020). Similar to cAMP, ERK can be activated at the plasma membrane or intracellular compartments by either G-proteins or β-arrestins (Eishingdrelo, 2013). The potent β-arrestin recruitment and signaling outcomes induced by compound 56 compared to compound 44 and serotonin (Sniecikowska et al., 2020), highlights the likelihood that endosomal signaling is an important component of the 5-HT_1A_ receptor response.

### 5-HT_2A_ Endocytosis

5-HT_2A_ receptor endocytosis is induced upon serotonin binding (Bhattacharyya et al., 2002) which can be abolished by over-expression of dominant-negative dynamin-1 in C6 glioblastoma cells (Nicole et al., 2002). The receptor endocytosis is inhibited by expression of dominant-negative dynamin-1 in HEK293 cells (Bhatnagar et al., 2001). 5-HT_2A_ co-localizes with clathrin in NIH 3T3 mouse fibroblast cell lines (Willins et al., 1998). Together these data suggest that 5-HT_2A_ endocytosis occurs via CME (Table 2; Figure 2B).

5-HT_2A_ appears to have cell-type variation in its requirement of β-arrestins for endocytosis. 5-HT_2A_ endocytosis is β-arrestin2 dependent in isolated mouse cortical neurons, mouse embryonic fibroblasts (Schmid et al., 2008) and rat C6 glioblastoma cells (Nicole et al., 2002). In HEK293 cells expressing rat 5-HT_2A_, serotonin induces dynamin-dependent endocytosis of 5-HT_2A_ and β-arrestin1 and 2 translocation to the plasma membrane, but expression of dominant negative β-arrestin1/2 does not alter 5-HT_2A_ endocytosis (Bhatnagar et al., 2001). Dominant-negative β-arrestin1 also does not affect rat 5-HT_2A_ receptor desensitization in HEK293 cells, consistent with the lack of effect on endocytosis, while it prevents 5-HT_2A_ receptor desensitization in C6 glioblastoma cells (Gray et al., 2001). GRKs do not significantly contribute to rat 5-HT_2A_ endocytosis in HEK293 or C6 cells, (Gray et al., 2001), yet expression of dominant negative GRK2 inhibited human 5-HT_2A_ endocytosis by 50%, and dominant negative β-arrestin by 70% in mouse AB1 cells (Bhattacharya et al., 2010). It is worth noting that in the Human Protein Atlas, 5-HT_2A_ is highly expressed in the brain, with very low levels of expression throughout most other tissues in the human body (Uhlén et al., 2015) while HEK293 cells do not express any 5-HT_2A_ whatsoever (Thul et al., 2017). Rat 5-HT_2A_ contains crucial sequence differences to human 5-HT_2A_ (Bhattacharya et al., 2010), and when expressed in HEK293 cells is in ways an artificial system that potentially lacks the requisite accessory factors that may be required for arrestin recruitment and endocytosis of the receptor. It is likely that in a physiological context, β-arrestins and GRK2 phosphorylation are a requirement for 5-HT_2A_ endocytosis following serotonin binding. Further study on human 5-HT_2A_ is required to better understand the role of GRFs in receptor internalization.

In addition to β-arrestins, PKC phosphorylation following serotonin binding is required for rat 5-HT_2A_ receptor endocytosis (Raote et al., 2013). Crucially, a wide range of 5-HT_2A_ receptor agonists induce differential phosphorylation of both rat and human 5-HT_2A_ and have differing requirements for PKC activity and β-arrestin recruitment (Figure 2B). Serotonin, dopamine, the hallucinogenic compound 2,5-Dimethoxy-4-iodoamphetamine (DOI) and anti-psychotic clozapine all induce rat 5-HT_2A_R endocytosis in HEK293 cells. Mutation of the Ser291 PKC phosphorylation site abolishes serotonin and DOI induced 5-HT_2A_ receptor endocytosis, but not that of dopamine and clozapine (Raote et al., 2013). LSD and DOI, but not the chemically related, non-hallucinogenic compounds lisuride and ergotamine, lead to PKC-dependent phosphorylation of human 5-HT_2A_ at Ser280 (Karaki et al., 2014). LSD and DOI induce a comparable signaling response to serotonin in both HEK293 cells and cortical neurons, while inducing minimal 5-HT_2A_ receptor internalization and β-arrestin2 interaction. In contrast, lisuride and ergotamine induce robust 5-HT_2A_ internalization and β-arrestin2 interaction, while minimally activating 5-HT_2A_ receptor signaling. Interestingly, mutation of the Ser280 PKC phosphorylation site abolishes 5-HT_2A_ receptor signaling in response to LSD and DOI treatment, but not serotonin treatment (Karaki et al., 2014), highlighting the differential signaling induced by serotonin and putatively therapeutic hallucinogenic compounds. Finally, a recent study by Hayata-Takano et al. (2021) identified that pituitary adenylate cyclase-activating polypeptide (PACAP) regulated the PKC and β-arrestin-dependent endocytosis of human 5-HT_2A_, but not 5-HT_1A_ or 5-HT_2C_, in HEK293 cells. In cortical neurons isolated from PACAP knockout mice, 5-HT_2A_ expression on the cell surface was increased, and these mice displayed a higher response to DOI compared to wild-type mice. Importantly, Karaki et al. (2014) and Hayata-Takano et al. (2021) reveal the importance of PKC phosphorylation and receptor internalization in the signaling response of 5-HT_2A_ to serotonin and hallucinogenic compounds.

Serotonin and other agonists induce differential endosomal sorting of 5-HT_2A_. Human 5-HT_2A_ is sorted into Rab11^+^ recycling endosomes *via* EEA1^+^ early endosomes following serotonin-induced internalization in HEK293 cells (Baldys and Raymond, 2011), indicating 5-HT_2A_ undergoes relatively “slow” recycling and resensitization. Complete recycling of all rat 5-HT_2A_ internalized into HEK293 cells with serotonin treatment takes ~2.5 h (Raote et al., 2013). DOI-induced 5-HT_2A_ endocytosis occurs in a comparable timeframe and magnitude to serotonin-induced endocytosis, yet 5-HT_2A_ recycling takes significantly longer (~7.5 h) with DOI treatment compared to serotonin (Raote et al., 2013) and occurs through an undefined pathway. DOI also induces near-identical levels of human 5-HT_2A_- mediated ERK phosphorylation as serotonin *in vivo* and in HEK293 cells (Schmid et al., 2008; Karaki et al., 2014). Despite this similar induction of ERK phosphorylation *in vivo*, β-arrestin KO mice stop head twitching in response to serotonin treatment, while those treated with DOI do not. DOI induced 5-HT_2A_ endocytosis is also only partially reduced in β-arrestin KO MEFs compared to serotonin (Schmid et al., 2008). Together, these studies indicate that the intracellular residence time of 5-HT_2A_ in endosomes induces different cellular or physiological outcomes in response to 5-HT_2A_ agonists across species, implicating endosomal signaling as an important effector of the 5-HT_2A_ receptor response.

Finally, the interaction between the corticotrophin-releasing factor (CRF) receptor and 5-HT_2A_ (Magalhaes et al., 2010) further demonstrates that specific signaling outcomes of 5-HT receptors are attractive targets for depression and anxiety treatments. In mouse cortical neuron cultures and HEK293 cells, treatment with the stress induced peptide CRF enhances serotonin-induced 5-HT_2A_ signaling. CRF and serotonin co-treatment resulted in higher inositol phosphate production than with serotonin treatment alone. Over-expression of dominant-negative Rab4, but not dominant negative Rab11, abolished the combined CRF and serotonin increase in inositol phosphate production (Magalhaes et al., 2010), indicating that rapid recycling of 5-HT_2A_ is required for sustaining 5-HT_2A_ signaling, at least in response to CRF in HEK293 cells. *In vivo*, administration of the 5-HT_2A_ agonist DOI or CRF each had no detrimental effects on mouse behavior however, co-administration of both compounds induced anxiety related behaviors. Together, the study by Magalhaes et al. (2010) further highlights the importance of the differential outcomes of 5-HT receptor signaling in depression and anxiety, and how endosomal trafficking modulates this signaling. A deeper understanding of 5-HT receptor endosomal signaling, 5-HT receptor endosomal trafficking, and inducing/limiting such signaling and trafficking are clearly important in the development of antidepressant and anxiolytic treatments targeting 5-HT receptors.

### Serotonin Transporter Endocytosis

The mechanism of SERT endocytosis is yet to be precisely defined, although it is likely that it occurs via CME (Figure 2C; Table 2). SERT endocytosis can be induced by serotonin (Jørgensen et al., 2014), or occurs constitutively (Rahbek-Clemmensen et al., 2014). In HEK293 cells and mouse neuronal CAD cells, constitutive SERT endocytosis is inhibited by expression of dominant-negative dynamin-1 (Rahbek-Clemmensen et al., 2014). In HEK293 cells and rat platelets, PKC phosphorylation of SERT induces endocytosis in the presence of serotonin (Qian et al., 1997; Jayanthi et al., 2005), indicating serotonin-induced SERT endocytosis is PKC-dependent. SERT endocytosis is also induced by serotonin in serotonergic dorsal raphe nucleus neurons and in HEK293 cells whereby serotonin-induced SERT endocytosis subsequently reduces serotonin uptake (Jørgensen et al., 2014). Endocytosis is clearly important in regulating serotonin uptake into the cell. Both WT SERT and a constitutively active SERT mutation (G56A) interact with components of CME in mouse models, indicating SERT endocytosis is clathrin-dependent *in vivo* (Quinlan et al., 2020). WT SERT interacts with the CME facilitators AP180, FCHO1, Eps15, and Reps2, as well as multiple dynamin-related components, and these interactions are decreased in the brains of mouse expressing the constitutively active SERT G56A mutation (Quinlan et al., 2020). This study therefore suggests clathrin-dependent endocytosis is required for endocytic downregulation of SERT *in vivo*.

As with SERT endocytosis, the endosomal sorting of SERT still requires precise definition (Figure 2C; Table 2). Constitutive endocytosis primarily targets SERT to the lysosome in HEK293 and CAD cells, although significant co-localization with the rapid recycling marker Rab4 is observed, and pharmacological inhibition of recycling reduces SERT cell surface levels (Rahbek-Clemmensen et al., 2014). SERT interacts with the Rab11 sorting mediator flotillin-1 in HEK293 cells and mouse brains (Reisinger et al., 2019; Quinlan et al., 2020), indicating that SERT may be able to undergo Rab11-dependent recycling *in vivo* and in cell models.

Induction of SERT endocytosis represents a primary mode of action of SSRIs. All SSRIs induce SERT endocytosis in serotonergic 1H11 cells (Kittler et al., 2010). Escitalopram and sertraline also induce endocytosis in stem cell generated serotonergic neurons (Matthäus et al., 2016). Mice treated with paroxetine and sertraline, show reduced SERT binding availability in the brain despite unaltered mRNA levels (Benmansour et al., 1999). With sertraline treatment, this was not due to the SERT binding site being occupied by the SSRI, rather, it was due to downregulation of transporter-independent transcription (Benmansour et al., 2002). Long term treatment of CACO-2 cells with fluoxetine reduces plasma membrane SERT levels without altering total protein levels (Iceta et al., 2007). All SSRIs induce SERT endocytosis and do not appear to regulate SERT transcription or translation, therefore it is highly plausible that SSRIs reduce SERT membrane levels *via* endocytosis.

SSRI-induced SERT endocytosis could also suggest that SERT uptake occurs via a non-CME mechanism. Sertraline is an established and potent dynamin-1 inhibitor (Otomo et al., 2008) which induces SERT endocytosis in serotonergic neurons (Matthäus et al., 2016) and in 1C11 cells (Kittler et al., 2010) despite it being reported that SERT endocytosis is dynamin-1 dependent. CME has never been reported to occur without dynamin, indicating that sertraline treatment may re-route SERT endocytosis *via* an alternate clathrin-independent endocytic pathway yet to be characterized (Figure 2C). A better understanding the SERT endocytic pathways will therefore enhance our understanding of how SSRIs act *in vivo* and at a cellular level, allowing for the identification of specific endosomal targets to modulate SERT levels at the plasma membrane therapeutically.

## Endocytic Dysregulation in Depression and Anxiety

Identification of directly causative genetic, transcriptomic, or proteomic changes associated with depression and anxiety has been difficult. Studies identifying biological changes in depression and anxiety may rely on self-reported diagnoses from large population databases, capturing broad depression and anxiety phenotypes (Thorp et al., 2021). Others use the relatively strict diagnosis of MDD, yet this still encapsulates multiple heterogenous depressive phenotypes. Depression and anxiety are also often comorbidities with other additional conditions adding to the complexity (Ormel et al., 2019). Studies are often performed on peripheral tissues due to the difficulty in accessing brain tissue (Wittenberg et al., 2020; Thorp et al., 2021), making it difficult to separate neurological and peripheral changes associated with depression and anxiety. A smaller number of studies are performed on post-mortem brain tissues however, patient numbers are very limited (Kang et al., 2012). Despite the heterogeneity in the way these association studies are performed, and consistent with our theory that endocytosis is an important biological factor in depression and anxiety, multiple single nucleotide polymorphisms (SNPs), transcriptional and proteomic changes relating to proteins have been associated with depression and anxiety. The following table documents the changes in proteins related to endocytosis that are also associated with depression and anxiety and details their roles in endocytosis and endosomal sorting (Table 3).

**Table 3:** Gene, transcript and protein changes associated with depression and anxiety.

**Protein**	**Change**	**Endocytic involvement**	**Tissue**	**Diagnosis**	**References**
PKC	Decreased protein expression	5-HT_2A_ and SERT phosphorylation for endocytosis	Brain	Depression	Pandey et al. (2021)
β-arrestin1 and 2	Decreased protein expression	Adaptor for 5-HT_1A_ (β-arrestin1) and 5-HT_2A_ (β-arrestin2) CME	Leukocytes	Depression, MDD	Avissar et al. (2004); Golan et al. (2013)
PACSIN3	SNP, functional change unknown	Membrane bending, dynamin recruitment	Blood (UK Biobank)	Depression, anxiety	Thorp et al. (2021)
DENND1A	SNP, functional change unknown	Connecting Rab35 endocytic compartments with CME	Genome-wide study	MDD	Wray et al. (2018)
DENND1B	Transcript downregulated	Required for endosome trafficking from cell periphery	Brain, blood	Depression	Dall'Aglio et al. (2021)
Flotillin-1	Protein upregulated	Rab11 endosomal recycling, interacts with SERT	Brain, periphery	MDD	Zhong et al. (2019)
EHD1	Overexpressed	Sorting between Rab5 and Rab11 endosomes for recycling	Brain	Depression	Yoshino et al. (2021)
RabGAP1L	SNP, functional change unknown	Inactivates Rab22, a negative regulator of endosomal recycling	Blood (UK Biobank)	Depression, anxiety	Thorp et al. (2021)
Rab4B	Protein expression decreased	Rapid recycling from sorting endosomes	Brain	Depression	Kang et al. (2012)
PICALM	Transcript overexpressed	Clathrin coat assembly	Peripheral blood mononuclear cells (PBMC)	Depression	Wittenberg et al. (2020)
Rac1	Transcript overexpressed	Actin remodeling for clathrin-independent endocytosis	PBMC	MDD	Wittenberg et al. (2020)
PACSIN2	Transcript overexpressed	Membrane bending, dynamin recruitment, potential involvement in FEME	PBMC	MDD	Wittenberg et al. (2020)
Rab5	Transcript overexpressed	Endosomal sorting regulator	PBMC	MDD	Wittenberg et al. (2020)
Rab7	Transcript overexpressed	Late endosomal regulator	PBMC	MDD	Wittenberg et al. (2020)
SNX27	Transcript overexpressed	Retromer complex component for recycling from Rab7 endosomes	PBMC	MDD	Wittenberg et al. (2020)

### Clathrin Endocytic Regulators and Adaptors

#### PKC

PKC protein expression is decreased in post-mortem brain samples of patients with depression (Pandey et al., 2021). PKC is activated by signaling through 5-HT_2A_ (Masson et al., 2012), and PKC phosphorylation of 5-HT_2A_ and SERT cytoplasmic C-termini are required for their internalization (Jayanthi et al., 2005; Raote et al., 2013). In patients with reduced PKC levels, 5-HT_2A_ and SERT endocytosis is likely to be reduced.

#### β-Arrestin

Reductions in β-arrestin1 levels correlate with depression severity in leukocytes of major depressive disorder patients, and antidepressant treatment increases β-arrestin1 levels in rat brains (Avissar et al., 2004). Further, antidepressant treatment rescues the reduction in both β-arrestin1 and 2 observed in leukocytes of major depressive disorder patients (Golan et al., 2013). Reduced β-arrestin levels in patients with depression would likely reduce both 5-HT_1A_ and 5-HT_2A_ endocytosis given their established interaction with β-arrestins (Della Rocca et al., 1999; Schmid et al., 2008), while β-arrestin rescue by antidepressant treatment would potentially restore 5-HT_1A_ and 5-HT_2A_ endocytosis rates.

#### PACSIN3

PACSIN3 SNPs are associated with depression and anxiety (Thorp et al., 2021). PACSIN3 is the least characterized member of the PACSIN/syndapin protein family of BAR-domain, SH3-domain containing proteins (Modregger et al., 2000). Each PACSIN family member interacts with dynamin, the actin remodeling protein N-WASP, and the phosphatase involved in clathrin coated pit assembly, synaptojanin-1. PACSIN3 over-expression inhibits the uptake of the clathrin-dependent cargo, transferrin (Modregger et al., 2000). The interaction of PACSIN3 with synaptojanin and dynamin indicates it may play a role in recruiting uncoating and scission machinery to the clathrin-coated pit, and the inhibitory effect of PACSIN3 over-expression may be due to the propensity for PACSIN proteins to self-oligomerize (Modregger et al., 2000), potentially sterically hindering dynamin and synaptojanin recruitment. It is unknown what the effect of PACSIN3 depletion on endocytosis is, but it seems plausible that a mutation reducing PACSIN3 function would have reductive effects on CME by reducing dynamin and synaptojanin recruitment.

#### PICALM

PICALM (or CALM) transcripts have been identified as upregulated in a meta-analysis by Wittenberg et al. (2020) consisting of 8 studies on peripheral blood mononuclear cells (PBMC) from patients diagnosed with MDD. PICALM interacts with clathrin heavy chain, the clathrin adaptor AP2 and facilitates the assembly of clathrin lattices, facilitating the progression of endosome formation (Tebar et al., 1999; Meyerholz et al., 2005). PICALM over-expression has a dominant negative effect on CME, preventing transferrin and epidermal growth factor receptor endocytosis (Tebar et al., 1999), while PICALM depletion also inhibits CME (Meyerholz et al., 2005), highlighting its critical role in the CME process.

#### DENND1

DENND1A SNPs are associated with depression (Wray et al., 2018), and the DENND1B transcript is downregulated in patients with depression (Dall'Aglio et al., 2021). DENND1A and B (or connecdenn1 and 2) are DENN domain containing proteins. The DENN domains of DENND1A and B act as guanine exchange factors for Rab35, activating Rab35 by facilitating GTP loading in the GTPase domain. DENND1A and B also interact with clathrin heavy and light chains, as well as the clathrin adaptor AP2 (Marat and McPherson, 2010). Rab35 recruits the 5' phosphatase ORCL to clathrin-coated pits, facilitating the PI(4,5)P_2_ catabolism required for scission of the clathrin-coated pit from the plasma membrane. DENND1A knockdown or dominant negative Rab35 over-expression result in endocytic cargoes being trapped in the cell periphery, consistent with incomplete clathrin-coated pit scission from the plasma membrane (Cauvin et al., 2016). DENND1B knockdown causes enlargement of early endosomes and aberrant intracellular trafficking of the clathrin-dependent endocytic cargo transferrin (Marat and McPherson, 2010), and DENND1B knockout and Rab35 knockdown reduces internalization of the T-cell receptor subunit CD3ε in T cells (Yang et al., 2016). DENND1A/B are therefore likely required for Rab35 activation for clathrin-coated pit scission from the plasma membrane and subsequent cargo vesicle incorporation into early endosomes.

### Endosomal Sorting Regulators

#### Rac1 and PACSIN2

Rac1 and PACSIN2 transcripts are upregulated in PBMCs from MDD patients (Wittenberg et al., 2020). Rac1-GTPase is involved in actin remodeling required for clathrin-independent endocytosis and Rac1 activation is required for FEME (Boucrot et al., 2015), dynamin-dependent, clathrin-independent endocytosis of the interlukin-2 receptor (Grassart et al., 2008), fluid-phase uptake by macropinocytosis (Fujii et al., 2013), and dynamin-independent, clathrin-independent endocytosis of the nicotinic acetylcholine receptor (Kumari et al., 2008). PACSIN2 is another member of the PACSIN/syndapin protein family of BAR-domain, SH3-domain containing proteins. Similar to PACSIN3, PACSIN2 is capable of interacting with N-WASP, synaptojanin-1 and dynamin, and PACSIN2 over-expression inhibits CME (Modregger et al., 2000). PACSIN2 is the PACSIN family member most enriched on FEME endocytic carries (Chan Wah Hak et al., 2018), implicating it in clathrin-independent endocytosis. PACSIN2 over-expression induces membrane tubulation at the plasma membrane of the cell that are positive for the clathrin-independent endocytic cargo cholera toxin, but not the CME cargo transferrin. PACSIN interacts with Rac1, inducing GTP hydrolysis and inactivation (de Kreuk et al., 2011). The Rac1 activation/deactivation cycle is required for proper progression of Rac1-dependent endocytic mechanisms (Fujii et al., 2013), suggesting that over-expression of both Rac1 and PACSIN2 could facilitate increased uptake by Rac1-dependent endocytic pathways such as FEME and macropinocytosis.

#### RabGAP1L

SNPs in RABGAP1L are associated with depression (Thorp et al., 2021). RabGAP1L is a GTPase activating protein responsible for hydrolyzing GTP to GDP in Rab22, inactivating it. RabGAP1L is recruited to PI(3)P-enriched early endosomes by the protein Ankyrin-B (Qu et al., 2016). PI(3)P-enriched early endosomes represent Rab5^+^ sorting endosomes that are responsible for cargo sorting for recycling and degradative fates (Redpath et al., 2020). Rab22 is present on a subset of these sorting endosomes, facilitating the recycling of both clathrin-dependent and independent endocytic cargoes (Weigert et al., 2004; Holloway et al., 2013). When RabGAP1L recruitment to Rab22^+^ sorting endosomes is disrupted by dominant negative Ankyrin-B expression, Rab22 accumulates on sorting endosomes and cargo recycling is disrupted (Qu et al., 2016), indicating RabGAP1L is required for cargo progression through sorting endosomes for subsequent recycling.

#### FLOT1

Flotillin-1 (or reggie-2) is upregulated in the brain and peripheral tissues of depression sufferers (Zhong et al., 2019). Flotillin-1 directly interacts with SERT (Reisinger et al., 2019), and the over-active SERT variant G56A has decreased interaction with flotillin-1 *in vivo* (Quinlan et al., 2020). Flotillin1 acts as an obligatory heterodimer with flotillin-2 (or reggie-1) (Solis et al., 2007). Together, flotilin1/2 mediate receptor and cargo sorting into the Rab11^+^ endocytic recycling network, regulating cargo entry into Rab5^+^ endosomes, facilitating delivery into Rab11^+^ endosomes for cargo recycling (Solis et al., 2013; Redpath et al., 2019). Consistent with flotillin-1 being a regulator of SERT intracellular trafficking, the dopamine transporter, which is closely related to SERT, is incorporated into flotillin-1-positive vesicles following PKC-induced endocytosis (Cremona et al., 2011). Flotillin-1 overexpression in patients with depression would be likely to facilitate SERT recycling, increasing plasma membrane levels and facilitate continued serotonin uptake into the cell.

#### EHD1

EHD1 is overexpressed in the prefrontal cortex of depression sufferers (Yoshino et al., 2021). EHD1 is a membrane of the FERARI sorting complex, which forms tubules from Rab5^+^ sorting endosomes to facilitate cargo transfer to Rab11+ recycling endosomes (Solinger et al., 2020). Further, EHD1 interacts with Rab11 sorting regulators flotillin1/2 (Solis et al., 2013), and is required for cargo transit throughout the entire Rab11^+^ recycling endosomal compartment (Lee et al., 2015). EHD1 over-expression in the brains of depression patients would be highly likely to facilitate increased receptor or transporter recycling.

#### Rab4B

Rab4B protein expression is decreased in the brains of depression sufferers (Kang et al., 2012). Rab4B interacts with the clathrin adaptor AP-1 and localizes to Rab5+ sorting endosomes, and over-expression enhances rapid transferrin recycling (Perrin et al., 2014). As Rab4A regulates the rapid recycling of the β2 adrenergic receptor, another GPCR internalized in a β-arrestin dependent manner (Yudowski et al., 2009), and Rab4B appears to be similar involved in rapid recycling of the transferrin receptor (Perrin et al., 2014), the Rab4 family may facilitate rapid GPCR recycling for continued receptor stimulation and recycling over sustained endosomal signaling.

#### Rab5, Rab7, and SNX27

Rab5, Rab7, and SNX27 transcripts are upregulated in PBMCs from MDD patients (Wittenberg et al., 2020). Rab5 is the predominant regulator of endosomal sorting. Rab5^+^ sorting endosomes mature to Rab7^+^ late endosomes, with cargoes that are not sorted for Rab4/Rab11-dependent recycling retained throughout this sorting to late endosomal transition (Rink et al., 2005; Redpath et al., 2020). Rab7^+^ late endosomes can then fuse with lysosomes, leading to cargo degradation. However, cargoes present in Rab7^+^ endosomes can be spared from degradation by recycling *via* the retromer/retriever recycling pathways, which bind cargoes in specific cellular circumstances, removing them from the Rab7^+^ late endosome and delivering them to the trans-Golgi network for recycling (Seaman, 2012; McNally et al., 2017).

SNX27 is a member of the sorting nexin family of cargo sorting regulators and plays an integral role in cargo sorting for the retromer complex (Temkin et al., 2011; McNally et al., 2017). SNX27 interacts with a wide range of proteins, including PDZ domains containing proteins such as GPCRs, targeting them for plasma membrane recycling (Lauffer et al., 2010; Temkin et al., 2011). 5-HT_2A_ contains a PDZ domain (Xia et al., 2003), and SERT interacts with PDZ-domain containing proteins (Chanrion et al., 2007), indicating both proteins may be able to be recovered from late endosomes by the retromer complex. Together, upregulation of Rab5 and Rab7 is likely to increase the trafficking flux though the sorting to late endosomal pathway, with SNX27 upregulation balancing this flux by increasing retromer-based recycling from Rab7^+^ late endosomes.

## Hypothesis: Impaired CME and Enhanced Endosomal Recycling Occurs in Depression and Anxiety

**Figure 3:**
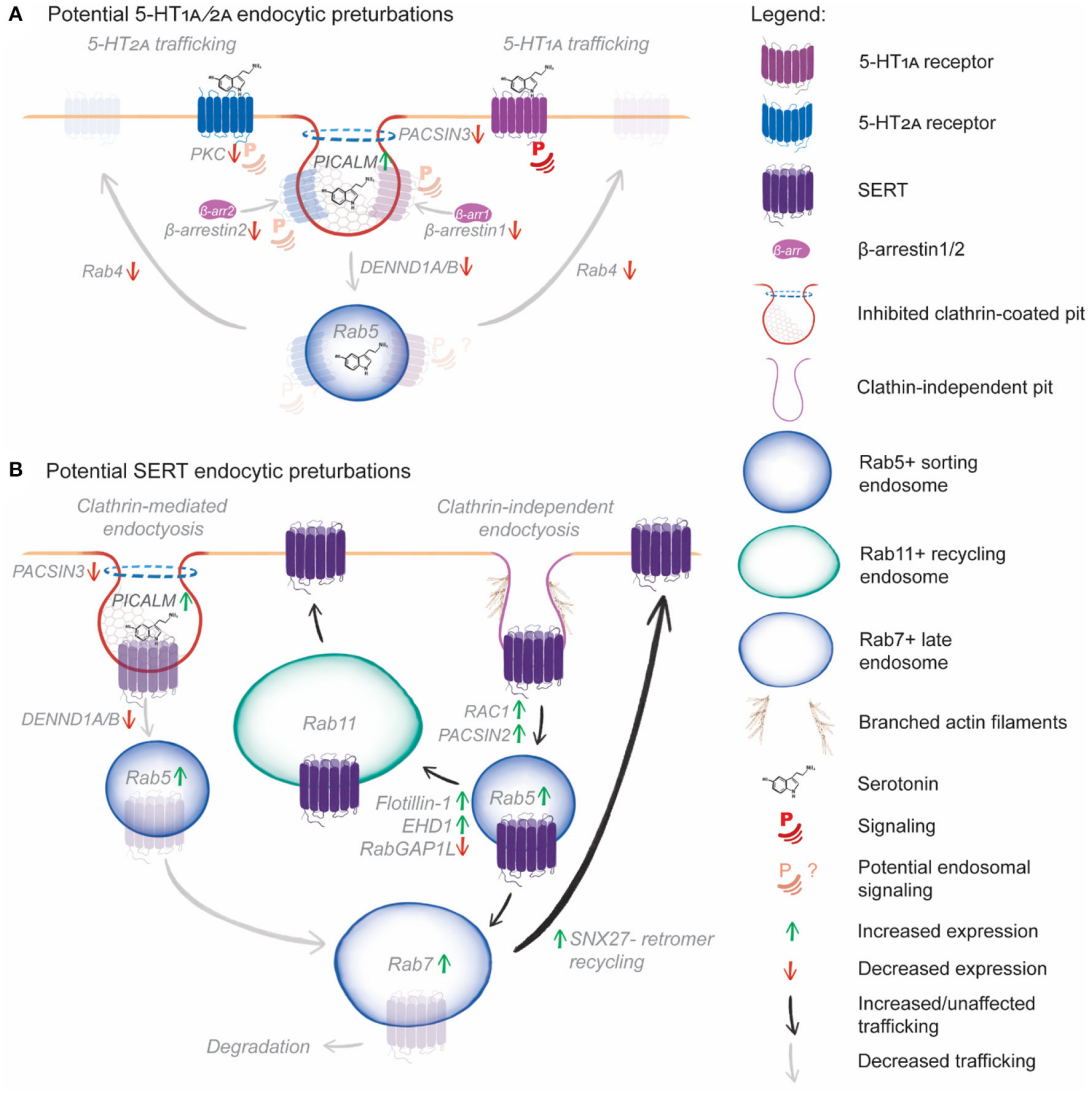
Hypothetical model of the effect of SNPs, transcript and protein expression changes on serotonin receptor and transporter endocytic trafficking. **(A)** Reduced PKC phosphorylation will reduce 5-HT_2A_ plasma membrane signaling and β-arrestin recruitment. Reduced β-arrestin1 and 2 levels will reduce CME of both 5-HT_1A_ and 5-HT_2A_. Further, increased PICALM expression inhibits clathrin-coated pit formation, and PACSIN3 mutations may impair dynamin recruitment, further reducing CME. DENND1A/B mutations could impair coupling of CME to Rab5^+^ sorting endosomes, reducing uptake, downstream receptor sorting and potential endosomal signaling. Finally, reduced Rab4B expression will prevent rapid recycling of 5-HT_1A_ and 5-HT_2A_, reducing the potential for rapid resensitization following serotonin-induced endocytosis. **(B)** Increased PICALM expression, PACSIN3 and DENND1A/B mutations are likely to impair CME of SERT. However, PACSIN2 and Rac1 over-expression could induce CIE of SERT by enhancing actin branching, leading to increased clathrin-independent carrier formation. Following CIE, increased Rab5 and Rab7, expression could increase early to late endosomal trafficking of SERT, following which increased SNX27 expression would salvage SERT from degradation and boost recycling. Alternately, increased EHD1 and flotillin expression would increase sorting of SERT from Rab5^+^ sorting endosomes to Rab11^+^ recycling endosomes, further enhancing SERT recycling.

The genetic, transcriptomic and proteomic changes associated with depression and anxiety detailed above (Table 3) all coalesce in an endosomal network where CME and Rab4-dependant rapid recycling are reduced, while clathrin-independent endocytosis, Rab11-dependant and retromer-dependent recycling are increased. We hypothesize that these changes contribute to the pathogenesis of depression and anxiety by disrupting the normal endocytic trafficking of serotonin receptors and transporter. While patients suffering from depression and anxiety will not possess every SNP, transcript or protein change, we speculate that any of these changes could result in decreased endocytosis of 5-HT_1A_ and 5-HT_2A_, disrupting their typical plasma membrane and endosomal signaling profiles (Figure 3A), or enhance clathrin-independent endocytosis and recycling of SERT or decrease SERT CME, increasing its levels at the plasma membrane (Figure 3B), which would deplete extracellular serotonin levels to further reducing serotonin signaling. Of note, for the following we assume that SNPs identified in the studies in Table 3 are deleterious for protein function, however this is yet to be experimentally determined and is a fascinating area of future research.

In our hypothetical model, PACSIN3 mutation, PACSIN2 overexpression, DENND1A mutation, DENND1B downregulation, and PICALM overexpression are likely to impair endocytic uptake by CME. This CME impairment could conceivably reduce the endocytosis of 5-HT_1A_, 5-HT_2A_ and SERT. Reduced β-arrestin levels would further impair 5-HT_1A_, 5-HT_2A_ endocytosis, while PKC downregulation would lead to reduced 5-HT_2A_ and SERT phosphorylation, reducing their endocytosis. Reduced 5-HT_1A_ endocytosis could lead to increased plasma membrane signaling, with reduced endosomal signaling, altering the cellular response to serotonin and other potential therapeutic agents. Reduced 5-HT_2A_ endocytosis could similarly impair 5-HT_2A_ endosomal signaling, while reduced PKC phosphorylation could lead to altered receptor phosphorylation patterns and diminished or aberrant responses to serotonin and hallucinogenic therapeutics. Rab4b downregulation would be likely to reduced rapid recycling of 5-HT_1A_ and 5-HT_2A_, which would impair receptor resensitization following endocytosis, further altering 5-HT receptor signaling outcomes. Reduced SERT CME would likely lead to increased plasma membrane SERT levels, leading to continued serotonin uptake from the extracellular space, reducing the availability of serotonin for signaling. Impaired CME is likely to reduce serotonin signaling through multiple mechanisms.

Clearly, there is a possibility that SERT undergoes clathrin-independent endocytosis upon treatment with the SSRI sertraline (Lau et al., 2008; Takahashi et al., 2010). In our hypothetical model, elevated PACSIN2 and Rac1 levels could increase SERT endocytosis by upregulating clathrin-independent endocytosis. Once endocytosed, upregulated flotillin-1 and EHD1 would increase SERT sorting for Rab11-dependent recycling. RabGAP1L mutations may conversely cause SERT accumulation in sorting endosomes, preventing delivery to recycling endosomes. Upregulated Rab5, Rab7, and SNX27 would salvage SERT from late endosomes, preventing degradation. Together, these mechanisms could act to redeliver endocytosed SERT to the plasma membrane, thus reducing the efficacy of SSRIs by ensuring plasma membrane levels of the transporter remain high.

## Conclusions

Endocytosis is clearly central to the regulation of serotonin signaling, SERT, the therapeutic response to hallucinogens, SSRI function and the function of novel antidepressant compounds. Here we present a hypothetical model of how SNPs, transcriptional and protein expression changes in patients with depression and anxiety could alter the endocytosis of serotonin receptors and SERT. This nascent area of neurobiological research highlights the potential impact of endocytosis in these disorders. Despite their apparent importance in mediating therapeutic effects, it is still unclear whether CME is required for SERT uptake, and 5-HT receptor and SERT-specific endocytic adaptors remain unidentified. Perhaps most startling is the lack of exploration of the effects of endosomal sorting on 5-HT-mediated serotonin signaling, despite clear evidence supporting endosomal signaling mechanisms for how each exert their cellular effects. It is abundantly clear that building a detailed understanding of the endocytic pathways of 5-HT receptors and SERT can enhance our understanding of the biological bases of depression and anxiety. A comprehensive understanding of antidepressants and hallucinogenic functions is critical in facilitating the development of the next generation of more effective antidepressants and anxiolytics and a deeper understanding of the genesis of these disorders.

## Data Availability Statement

The original contributions presented in the study are included in the article/supplementary material, further inquiries can be directed to the corresponding author/s.

## Author Contributions

ND and GR wrote the manuscript. ND prepared the figures. GR conceived of the manuscript. All authors contributed to the article and approved the submitted version.

## Conflict of Interest

The authors declare that the research was conducted in the absence of any commercial or financial relationships that could be construed as a potential conflict of interest.

## Publisher's Note

All claims expressed in this article are solely those of the authors and do not necessarily represent those of their affiliated organizations, or those of the publisher, the editors and the reviewers. Any product that may be evaluated in this article, or claim that may be made by its manufacturer, is not guaranteed or endorsed by the publisher.
